# Mental Health Issues and 24-Hour Movement Guidelines–Based Intervention Strategies for University Students With High-Risk Social Network Addiction: Cross-Sectional Study Using a Machine Learning Approach

**DOI:** 10.2196/72260

**Published:** 2025-06-13

**Authors:** Lin Luo, Junfeng Yuan, Chen Xu, Huilin Xu, Haojie Tan, Yinhao Shi, Haiping Zhang, Haijun Xi

**Affiliations:** 1 School of Physical Education, Guizhou Normal University, University Town, Siya Road, Huaxi District, Guiyang, 550025, China, 86 86751983; 2 Key Laboratory of Brain Function and Brain Disease Prevention and Treatment of Guizhou Province, Guiyang, China

**Keywords:** social network addiction, mental health, university students, 24-hour movement guidelines, intervention strategies

## Abstract

**Background:**

The exponential growth of digital technologies and the ubiquity of social media platforms have led to unprecedented mental health challenges among college students, highlighting the critical need for effective intervention approaches.

**Objective:**

This study aimed to explore the relationship between meeting the 24-hour movement guidelines (24-HMG) health behavior combinations and the risk of social network addiction (SNA) as well as mental health issues among university students. It further sought to compare differences in mental health indicators and SNA levels across various risk groups and adherence patterns, and to identify the optimal 24-HMG health behavior intervention strategies for students at high risk of SNA.

**Methods:**

This cross-sectional study recruited a total of 12,541 university students from the university town of Guizhou Province as participants. Data were collected through standardized questionnaires, including the Chinese version of Social Network Addiction Scale for College Students (SNAS-C), the adult attention-deficit/hyperactivity disorder (ADHD) self-report scale (ASRS), and the Chinese version of the *Diagnostic and Statistical Manual of Mental Disorders, Fifth Edition* (DSM-5) Self-Report Level 1 Cross-Cutting Symptom Measure for Adults (DSM-5 CCSM), among others. The primary analytical method used was the random forest model, which was used to explore the relationship between different 24-HMG behavior combinations and mental health variables among student at high-risk of SNA. In addition, the study aimed to identify the optimal 24-HMG health behavior intervention strategies for this high-risk group.

**Results:**

Participants in the meeting none group exhibited the highest SNA scores (57.98), which declined progressively with greater adherence. Among single-guideline groups, meeting physical activity (PA; 53.07) and meeting sedentary time (ST; 52.72) showed similar scores. Further reductions were seen in meeting PA+ST (49.68), meeting sleep (48.44), and meeting ST+sleep (44.75), with the lowest in meeting PA+ST+sleep. Approximately 6% of the variance in SNA was attributable to differences in adherence patterns (η²=0.06). Students meeting all three 24-HMG components—PA, sleep, and ST—demonstrated the strongest protection against attention deficit, depression, and anxiety. All 24-HMG behaviors were inversely associated with mental health symptoms, except academic satisfaction, which was positively correlated. Random forest modeling identified meeting sleep+ST as the most impactful for mania (0.4491), sleep disturbance (0.4032), personality (0.3924), and dissociation (0.3832). Meeting ST alone showed the strongest effects on substance (0.6176) and alcohol use (0.6597). Depression was influenced by meeting sleep+ST (0.2053), meeting PA+ST+sleep (0.1650), and meeting PA+ST (0.1634). The model achieved high accuracy for ASRS (0.912; *F*_1_-score=0.927), with robust predictions for substance use (*F*_1_-score=0.873) and mania (*F*_1_-score=0.836).

**Conclusions:**

Adherence to the health behaviors recommended by the 24-HMG can significantly improve the mental health outcomes of university students at high risk for SNA. The findings of this study support the development of mental health intervention strategies for students at high-risk of SNA based on the 24-HMG framework.

## Introduction

### Background

Social network addiction (SNA) is an emerging behavioral addiction characterized by excessive dependence on social network platforms, which significantly impairing an individual’s daily social functioning. In recent years, with the rapid proliferation of smartphones and internet technologies, SNA has rapidly proliferated globally, particularly among university students. Previous studies have indicated that university students, who face academic pressures, strong social interaction needs, and a high level of acceptance of new media technologies, are at higher risk of developing SNA [1,2]. An international survey reported that approximately 30% of university students experience varying degrees of SNA [3].

SNA not only directly disrupts an individual’s daily life functioning but is also closely related to a variety of mental health issues. A large body of empirical research has demonstrated a significant positive correlation between SNA and anxiety, depression, and personality disorders [2,4]. For instance, a study by Zhang et al [5] on Chinese university students found that individuals classified as high-risk for SNA exhibited significantly higher levels of anxiety and depressive symptoms compared to low-risk groups. In addition, SNA is closely associated with decreased sleep quality, impaired academic performance, and social dysfunction [6,7]. Recent studies further have revealed that SNA may exacerbate mental health issues by increasing loneliness, lowering self-esteem [2,8], and promoting negative social comparisons and cyberbullying [9,10]. Therefore, understanding the pathological mechanisms of SNA and exploring effective intervention strategies have become important research topics in the field of health psychology.

Although Griffiths’ 6D model of behavioral addiction (salience, tolerance, mood modification, relapse, withdrawal, and conflict) provides a solid foundation for the theoretical conceptualization of SNA [11-13], there are important differences between SNA and other types of internet addiction (eg, internet gaming disorder or compulsive internet use) in terms of specific cognitive and emotional mechanisms. Specifically, SNA involves more self-concept and self-presentation processes [14,15], as well as social anxiety, loneliness, and other specific social emotional processing mechanisms [16]. Moreover, recent neuroscience research has started to uncover the potential neurobiological basis of SNA. Structural magnetic resonance imaging (MRI) studies have begun that individuals with SNA exhibit significantly reduced gray matter volumes in the amygdala, ventral striatum, and orbitofrontal cortex [17,18]; functional MRI studies further indicate weakened functional connectivity between the frontal eye field and dorsolateral prefrontal cortex in individuals with SNA, suggesting impaired executive control and attention regulation [19]. Similar to other forms of addiction, the midbrain-limbic pathway and the prefrontal-striatal loop related to the dopamine system may play a key role in the reward sensitivity and impulsivity in SNA—reward sensitivity and impulsivity associated with SNA [20]. Notably, the unique functional alterations in the anterior cingulate cortex in SNA may distinguish it from other addiction types [17]. However, longitudinal studies that clarify the causal relationship between excessive social network use and brain function changes remain lacking [18] , and future research needs to strengthen the exploration of this mechanism.

Furthermore, existing evidence suggests that inappropriate use of the internet and social media among adolescents and young adults may reinforce the risk of SNA through complex interactions with lifestyle factors. On the one hand, numerous studies have found that SNA is closely associated with decreased PA, increased sedentary behavior, and sleep disorders [21-23]. On the other hand, regular PA has been shown to effectively buffer the adverse effects of SNA on depressive symptoms [24]. These studies suggest that interventions promoting a healthy lifestyle may effectively reduce the risk of SNA.

Recently, the World Health Organization’s 24-hour movement guidelines (24-HMG) have provided a systematic framework for improving individual health behaviors and mental health [25]. The 24-HMG recommend that adults engage in at least 150 minutes of moderate-intensity PA per week, reduce ST, and ensure 7‐9 hours of quality sleep per night. Empirical studies have shown that individuals who meet the 24-HMG recommendations for healthy behaviors exhibit better mental health, higher life satisfaction, and improved social functioning [26-28]. Recent research on adolescents further indicates that individuals who do not meet the 24-HMG recommendations for healthy behaviors are more likely to develop internet addiction behaviors [29], highlighting the potential of 24-HMG adherence in SNA interventions.

The mechanisms through which meeting the 24-HMG mitigates SNA risk may involve multiple pathways, such as circadian rhythm synchronization, cognitive fatigue reduction, and modulation of the dopamine reward system. Specifically, regular sleep and PA can stabilize the secretion rhythms of melatonin and cortisol, promoting emotional regulation and self-control [30]; simultaneously, regular PA and reduced sedentary behavior can effectively activate prefrontal cortex functions, enhancing attention and cognitive control [31,32]; in addition, PA naturally activates the dopamine system, reducing individuals’ excessive reliance on immediate social rewards (eg, social media interactions) [33].

However, current research still has notable limitations. First, existing studies have not fully explored the precise intervention effects of different activity combinations on specific mental health issues [34,35]; second, there is still a lack of research on personalized intervention strategies for university students at high risk of SNA [6,9]. Therefore, understanding the relationship between different 24-HMG adherence patterns and mental health, as well as establishing personalized intervention plans for high-risk populations, has significant theoretical and practical implications.

### Objectives

Based on the above background, this study aims to address the core questions shown in Textbox 1:

Textbox 1. **Research questions**

- Are there differences in mental health indicators and 24-HMG adherence patterns between university students with high-risk and low-risk of SNA?
- Are there differences in SNA levels between university students with different 24-HMG adherence patterns?
- Are there differences in mental health indicators among university students with different 24-HMG adherence patterns?
- For university students with high-risk of SNA, which 24-HMG adherence pattern shows the optimal intervention effect on improving specific mental health issues?

## Methods

### Participants and Procedure

This study used survey data collected from college students in the university town of Guizhou Province, China, between September and October 2024. A combination of internet-based and offline sampling methods was used. For internet-based sampling, participants were recruited through university-based social media platforms and email lists, while offline sampling involved stratified random selection of classrooms across different faculties. This approach collected data from 12,766 university students across 12 provinces, municipalities, and autonomous regions, including Guizhou, Guangxi, Chongqing, Sichuan, Beijing, Jiangsu, Shandong, Henan, Heilongjiang, and others. The initial response rate was approximately 85%, with 2250 students declining participation.

During the data preprocessing phase, 225 participants who had been clinically diagnosed with mental disorders were excluded to enhance the data quality and the reliability of the research conclusions. Ultimately, 12,541 valid questionnaire responses from undergraduate students were included in the analysis. All study participants confirmed having experience using social network platforms (such as Weibo, WeChat, and Xiaohongshu), meeting the inclusion criteria for this study.

### Ethical Considerations

Before the survey, all participants were thoroughly informed about the study’s purpose, principles of data confidentiality and anonymity, voluntary participation, and the right to withdraw at any time. To ensure informed consent, participants were informed that no material compensation would be provided for their participation. For offline participants, written consent was obtained after they fully understood the relevant information, while online participants were required to check the option “I have read the above information and agree to participate in this study” before proceeding to the questionnaire page.

The study protocol was approved by the Academic Ethics Committee of Guizhou Normal University (Approval No.: 20230300005), ensuring ethical compliance throughout the research process.

### Measures

#### Demographic Characteristics

All participants were asked about basic information, including gender, age, and academic year.

#### Social Network Addiction Scale

The Social Network Addiction Scale (SNAS) was developed by Shahnawaz et al [12] based on Griffiths’ 6-factor model of addictive behavior [4], aiming to assess social media addiction across 6 core dimensions: salience, mood modification, tolerance, withdrawal symptoms, conflict, and relapse. The original scale consists of 21 items, rated on a 5-point Likert scale (1=strongly disagree, 5=strongly agree). The Chinese version of the SNAS (SNAS-C) used in this study was revised and localized by Bi et al [13] based on the original scale. The revised SNAS-C maintains the same structure and dimensions as the original version and has demonstrated high reliability and validity in preliminary studies [13].

In this study, the overall Cronbach α for the SNAS-C was 0.958, indicating very high internal consistency. In addition, the Cronbach α values for the low-risk and high-risk groups of SNA were 0.957 and 0.951, respectively, further validating the reliability of the scale across different risk groups. Therefore, the SNAS-C is considered an effective tool for accurately measuring and assessing the level of social network addiction among college students. Based on the risk classification criteria for SNA proposed by Bi et al [13], this study categorized students with SNAS-C scores greater than 58 as the high-risk group and those with scores less than 58 as the low-risk group.

#### Adult Attention-Deficit/Hyperactivity Disorder Self-Report Scale

The adult attention-deficit/hyperactivity disorder (ADHD) self-report scale (ASRS), developed by the World Health Organization (WHO), is a standardized psychometric tool designed to assess core symptoms of ADHD in adult populations [36]. The ASRS consists of 18 items divided into 2Ds: inattention and hyperactivityand impulsivity. Each item is rated on a 5-point Likert scale (0=never, 4=very often), reflecting the individual’s behavioral performance over the past 6 months. The scale demonstrates good reliability and validity, with internal consistency coefficients (Cronbach α) typically exceeding 0.80 and high correlation with clinical diagnostic outcomes [36]. Due to its brevity and ease of use, the ASRS is widely applied in clinical screening, epidemiological surveys, and individual self-assessment, serving as an essential tool for early identification and intervention of ADHD. This study used the Chinese version of the ASRS [37], which has been validated for use in the Chinese population and demonstrates good reliability and validity. In this study, the Cronbach α for the ASRS was 0.954, indicating high reliability of the scale in the research context.

#### *Diagnostic and Statistical Manual of Mental Disorders, Fifth Edition* Self-Report Level 1 Cross-Cutting Symptom Measure for Adults

This study used the *Diagnostic and Statistical Manual of Mental Disorders, Fifth Edition* (DSM-5) Self-Report Level 1 Cross-Cutting Symptom Measure for Adults (DSM-5 CCSM) to assess participants’ psychiatric symptoms over the past 2 weeks [38]. The scale includes 23 items covering 13 psychopathological dimensions: depression (items 1‐3), mania (items 4‐5), anxiety (items 6‐8), somatic symptoms (items 9‐10), suicidal ideation (item 11), psychotic symptoms (items 12‐13), sleep problems (item 14), memory (item 15), obsessive-compulsive symptoms (items 16‐17), dissociative symptoms (item 18), personality functioning (items 19‐20), and substance use (items 21‐23). Each item is rated on a 5-point Likert scale (0=“none, not at all” to 4=“severe, nearly every day”), reflecting the severity of symptoms experienced by participants over the past 2 weeks. The scale has been validated in the Chinese population, with a Cronbach α coefficient of 0.89, indicating good internal consistency [37]. Therefore, the Chinese version of the DSM-5 CCSM was used as a reliable tool for measuring psychiatric symptoms in this study.

### Academic Stress and Academic Satisfaction

This study used a self-designed questionnaire to measure academic stress and academic satisfaction [39]. Academic stress was assessed through the question, “How much pressure do you feel from your current academic tasks?” using a 5-point Likert scale (1=“no pressure at all,” 5=“very high pressure”). Academic satisfaction was assessed through the question, “How satisfied are you with your current academic experience?” also using a 5-point Likert scale (1=“very dissatisfied,” 5=“very satisfied”). The measurement method was adapted from Cohen et al’s [40] stress assessment framework and Diener et al’s [41] satisfaction measurement approach to ensure the validity and reliability of the measured variables.

### 24-HMG Variables

This study used the Canadian 24-HMG as a reference standard [25] . The framework of these guidelines includes three core components: (1) PA guidelines, (2) ST guidelines, and (3) sleep guidelines. The measurement of these variables was based on self-report questionnaires designed in our previous studies [42-44], which comprehensively assessed adolescents’ PA frequency, duration of each activity, daily ST (including screen time), and self-reported sleep duration.

According to the 24-HMG standards, meeting the PA guideline is defined as engaging in at least 60 minutes of moderate-to-vigorous PA daily; meeting the ST guideline is defined as having no more than 180 minutes of screen time per day and no more than 8 hours of total ST; meeting the sleep guideline is defined as having 7 to 9 hours of sleep per night. The adherence to each guideline was coded as a binary variable: meeting the guideline (following) was coded as 1, and not meeting the guideline (not following) was coded as 0.

Based on adherence to the three guidelines, a mutually exclusive categorical variable was further constructed to represent the 24-HMG meeting patterns, which included the following 8 combinations:meeting none, meeting only one guideline (meeting PA, meeting ST, and meeting sleep), meeting any 2 guidelines simultaneously (meeting PA+ST, meeting PA+sleep, meeting ST+sleep), and meeting all 3 guidelines simultaneously (meeting PA+ST+sleep).

### Statistical Analysis

This study used a variety of statistical methods to analyze the data. First, independent samples *t* tests were used to compare differences in mental health indicators between different SNA risk groups, with effect sizes measured using Cohen *d*, where 0.2 indicates a small effect, 0.5 a medium effect, and 0.8 a large effect [45]. Second, *χ*^2^ tests were conducted to analyze the relationship between SNA risk and adherence to the 24-hour movement guidelines, with effect sizes assessed using Cramer V, where 0.1 indicates a small effect, 0.3 a medium effect, and 0.5 a large effect [46].

To further explore the relationship between the 24-HMG and SNA scores, one-way ANOVA was used, with effect sizes measured using η² (eta squared), where 0.01 indicates a small effect, 0.06 a medium effect, and 0.14 a large effect [47]. Subsequently, multiple regression analysis was used to build statistical models, controlling for confounding variables such as age, gender, and academic year, to examine the relationships between adherence to different activity guidelines (eg, PA, sleep duration, and screen time) and various mental health issues (eg, anxiety, depression, and stress). The explanatory power of the regression models was evaluated using adjusted *R*², and standardized regression coefficients (*β*) along with their significance levels were reported [48].

To identify optimal lifestyle intervention strategies for university students at high risk of SNA, a regression prediction model was constructed using the random forest algorithm [49]. To ensure data integrity, all features and target variables were uniformly converted into numerical values. A complete-case analysis was adopted to exclude samples with missing key variables, thereby enhancing the robustness of the results. The overall proportion of missing data was only 0.02%, randomly distributed without systematic patterns. According to established statistical guidelines, such low and random missingness is unlikely to introduce substantial bias; thus, imputation was not performed to avoid potential estimation bias.The dataset was randomly divided into training and testing sets in an 80:20 ratio. To eliminate the influence of varying feature scales, all input variables were standardized using the standard scaler method (mean=0, SD=1). Feature selection was conducted solely on the training set by combining Gini importance and mutual information to identify variables with substantial predictive value. The random forest model was trained on the training data, and key hyperparameters (eg, number of trees, maximum depth, and minimum samples for split) were optimized via 10-fold cross-validation and grid search. To mitigate overfitting, constraints were applied to the maximum tree depth and the number of features considered per tree. Model performance was evaluated on the testing set using the coefficient of determination (*R*²) as the primary regression metric, providing a comprehensive measure of predictive accuracy and generalizability. The final model yielded feature importance scores, which were visualized using a heatmap derived from the feature importance matrix to illustrate the contribution of each variable to risk prediction. These findings offer both a theoretical foundation and empirical evidence to support targeted intervention design.

All statistical analyses were performed using SPSS (version 26.0; IBM Corp) and Python (version 3.8; Python Software Foundation), with statistical significance defined as *P*<.05.

## Results

### Sample Characteristics

In this study, 12,541 university student participants were divided into a group with low-risk of SNA (n=6242) and a group with high-risk of SNA (n=6299). The average age of the group with low-risk of SNA was 19.80 years (SD 1.82), while the average age of the group with high-risk of SNA was 19.48 years (SD 1.42). In terms of gender distribution, the group with low-risk of SNA consisted of 49.78% (3107/6242) female participants and 50.22% (3135/6242) male participants, whereas the group with high-risk of SNA had a significantly lower proportion of female participants, with only 39.25% (2470/6299), and 60.75% (3823/6299) male participants.

In terms of mental health and behavioral characteristics, the group with high-risk SNA had significantly higher scores on the SNAS-C compared to the low-risk SNA group (mean 66.20, SD 7.34 vs mean=38.43, SD 11.89, *P*<.001), and also scored significantly higher on the adultASRS and several mental health dimensions (*P*<.001). The results indicated a large effect size for the difference in SNAS-C scores (Cohen *d*=−2.81), with an absolute value far exceeding the traditional threshold for a large effect (Cohen *d* ≥0.80), highlighting the excellent discriminative validity of this scale for stratifying SNA risk.

In the medium effect size range (0.50≤d<0.80), the group with high-risk of SNA showed clinically significant differences in core psychopathological dimensions, such as attention deficit symptoms (ASRS, Cohen *d*=−0.69), obsessive-compulsive symptoms (Cohen *d*=−0.58), anxiety (Cohen *d*=−0.58), and depression (Cohen *d*=−0.56). Notably, academic satisfaction was the only positively correlated indicator (Cohen *d*=0.58). Small effect size indicators (0.20≤d<0.50) revealed relatively weaker but statistically significant associations in the group with high-risk of SNA for substance use (alcohol: Cohen *d*=−0.31; tobacco: Cohen *d*=−0.20), sleep disorders (Cohen *d*=−0.50), and psychotic symptoms (Cohen *d*=−0.47), with all comparisons showing *P*<.001.

In addition, in terms of health behaviors, the group with high-risk of SNA had significantly lower adherence rates to the PA, sleep duration, and sedentary behavior guidelines compared to the group with low-risk of SNA (*P*<.001). Specifically, the adherence rate to the PA guideline in the high-risk group was 11.97% (753/6299), significantly lower than the 18.57% (1159/6242) in the low-risk group (Cramer V=0.10); regarding sleep duration, the compliance rate for the high-risk group was only 7.96% (501/6299), much lower than the 17.11% (1068/6242) in the low-risk group (Cramer V=0.13); and for sedentary behavior, the percentage of participants in the low-risk group failing to meet the guideline was 77.23% (4821/6242), higher than the 70.44% (4433/6299) in the high-risk group (Cramer V=0.10). Detailed sample characteristics are presented in Table 1.

**Table 1.** Demographic, mental health, and behavioral comparisons between university students with low and high SNA risk . Categorical variables are expressed as n (%), and continuous variables are expressed as mean (SD). For categorical variables, *P* values were obtained using the *χ*^2^ test; for continuous variables between two groups, *P* values were obtained using the independent samples *t* test; and for continuous variables among three groups, *P* values were obtained using one-way ANOVA.

Characteristic	Total (n=12541）	Low risk SNA (n=6242）	High risk SNA (n=6299）	*P* value	Cohen *d* or Cramer V
Age in years, mean (SD)	19.64 (1.64）	19.80 (1.82）	19.48 (1.42）	<.001	0.19
Sex, n (%)				<.001	0.11
Female	6962 (55.51）	3107 (49.78）	2470 (39.25）		
Male	5579 (44.49）	3135 (50.22）	3823 (60.75）		
Grade, n (%)				<.001	0.08
Freshman year	7151 (57.02）	3812 (61.07）	3339 (53.01）		
Sophomore year	4978 (39.70）	2220 (35.57）	2758 (43.75）		
Junior year	412 (3.28）	208 (3.33）	204 (3.19）		
SNAS-Cs^a^, mean (SD)	52.37 (17.03）	38.43 (11.89）	66.20 (7.34）	<.001	-2.81
ASRSs^b^, mean (SD)	23.5 (11.47）	19.72 (11.41）	27.25 (10.25）	<.001	-0.69
Substance use, mean (SD)	0.14 (0.45）	0.09 (0.36）	0.20 (0.53）	<.001	-0.25
Tobacco use, mean (SD)	0.31 (0.74）	0.25 (0.69）	0.37 (0.77）	<.001	-0.20
Alcohol use, mean (SD)	0.27 (0.59）	0.18 (0.50）	0.36 (0.66）	<.001	-0.31
Personality, mean (SD)	1.03 (1.40）	0.66 (1.17）	1.39 (1.51）	<.001	-0.55
Dissociation, mean (SD)	0.49 (0.70）	0.30 (0.58）	0.67 (0.76）	<.001	-0.55
OCD^c^, mean (SD)	1.16 (1.39）	0.77 (1.19）	1.55 (1.47）	<.001	-0.58
Memory, mean (SD)	0.63 (0.78）	0.44 (0.68）	0.83 (0.82）	<.001	-0.51
Sleep disturbance, mean (SD)	0.73 (0.82）	0.53 (0.73）	0.93 (0.86）	<.001	-0.50
Psychotic tendency, mean (SD)	1.11 (1.73）	0.71 (1.39）	1.50 (1.92）	<.001	-0.47
Suicidal tendency, mean (SD)	0.32 (0.63）	0.20 (0.51）	0.45 (0.71）	<.001	-0.40
Somatic, mean (SD)	1.27 (1.35）	0.90 (1.18）	1.63 (1.41）	<.001	-0.56
Anxiety, mean (SD)	2.34 (2.06）	1.76 (1.87）	2.91 (2.08）	<.001	-0.58
Mania, mean (SD)	1.6 (1.37）	1.29 (1.30）	1.89 (1.37）	<.001	-0.45
Anger, mean (SD)	0.81 (0.77）	0.61 (0.70）	1.01 (0.78）	<.001	-0.54
Depression, mean (SD)	1.74 (1.46）	1.34 (1.33）	2.13 (1.47）	<.001	-0.56
Academic stress, mean (SD)	1.47 (0.96）	1.23 (0.97）	1.69 (0.90）	<.001	-0.49
Academic satisfaction, mean (SD)	3.68 (0.89）	3.93 (0.90）	3.43 (0.80）	<.001	0.58
Physical activity, n (%)				<.001	0.10
Not following	10629 (84.75）	5083 (81.43）	5540 (88.03）		
Following	1912 (15.25）	1159 (18.57）	753 (11.97）		
Sleep time, n **(%)**				<.001	0.13
Not following	10972 (87.49）	5174 (82.89）	5792 (92.04）		
Following	1569 (12.51）	1068 (17.11）	501 (7.96）		
Sedentary time, n **(%)**				<.001	0.10
Not following	9258 (73.82）	4821 (77.23）	4433 (70.44）		
Following	3283 (26.18）	1421 (22.77）	1860 (29.56）		

a SNAS-C: Chinese version of the Social Network Addiction Scale.

b ASRS: attention-deficit/hyperactivity disorder self-report scale.

c OCD: Obsessive–compulsive disorder.

### Meeting to 24-HMG Guidelines

Among the 12,541 study samples （Multimedia Appendix 1), the highest proportion of participants adhered solely to the ST guideline, at 58.2% (7305/12541). In contrast, the proportions of participants adhering only to the PA guideline (“Meeting PA”) and the sleep guideline (“Meeting Sleep”) were very low, at 3.2% (404/12541) and 3.0% (377/12541), respectively. Only 1.6% (200/12541) of participants simultaneously adhered to both the PA and sleep guidelines (“Meeting PA+Sleep”), while 7.7% (961/12541) followed both the PA and ST guidelines (“Meeting PA+ST”). In addition, 5.1% (645/12541) adhered to both the sleep and ST guidelines (“Meeting ST+Sleep”). Finally, 2.8% (347/12541) of participants met all 3 guidelines for PA, sleep, and ST (“Meeting PA+ST+Sleep”).

### Comparison of SNAS-C Scores Among University Students With Different Meeting Patterns to the 24-HMG

**Figure 1.**
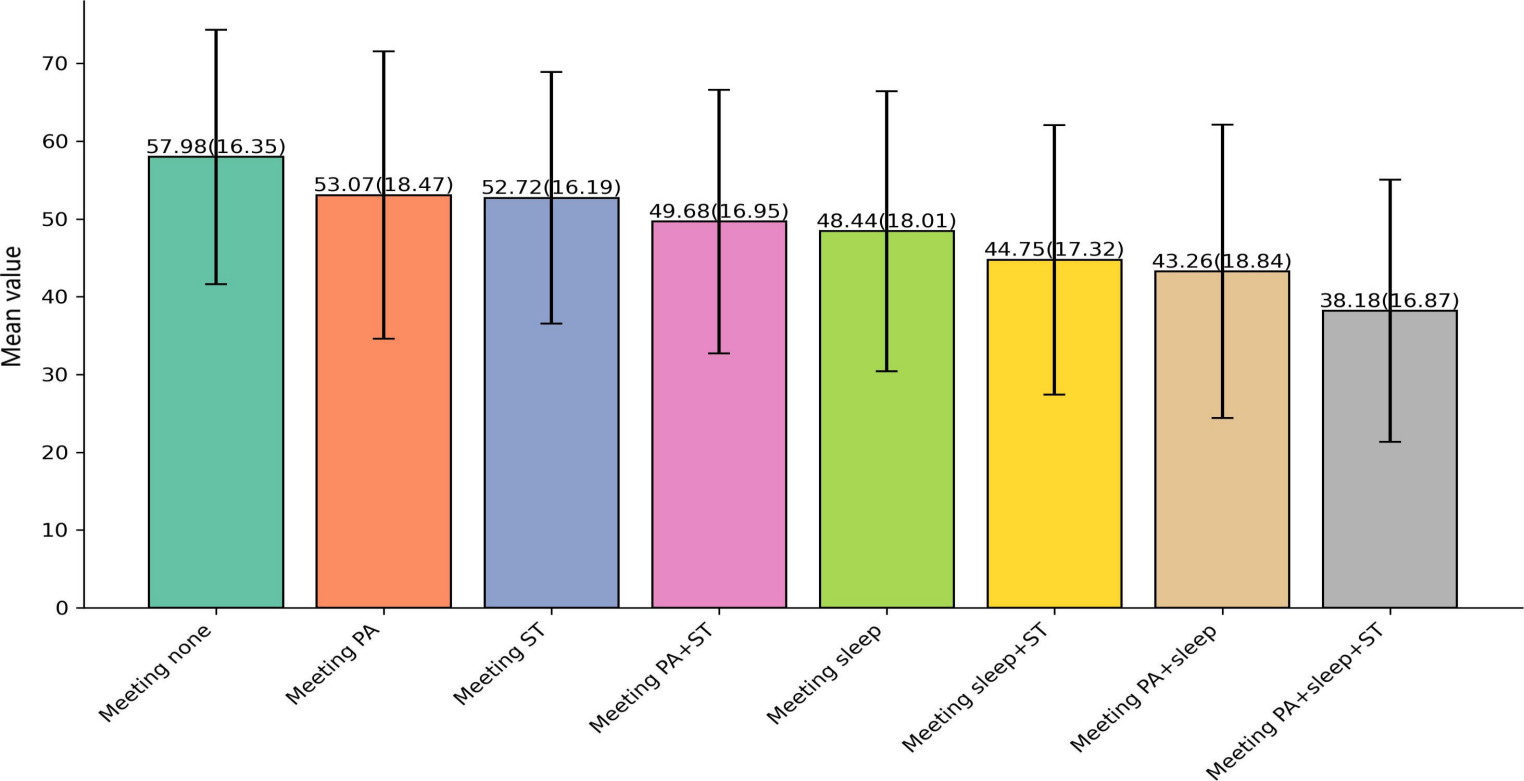
Impact of Different 24-HMG meeting patterns on social network addiction scores (SNAS-C).(The numbers above the bars represent the mean value [SD]). T: physical activity; ST: sedentary time.

Figure 1 and Multimedia Appendix 2 illustrate the impact of different activity participation levels on the total SNA score (SNAS-C). The “Meeting None” group, which did not meet any guidelines, had the highest average score of 57.98. As the number of met 24-HMG guidelines increased, the SNA scores showed a gradual decline. The average scores for the groups meeting only the PA guideline (Meeting PA) and only the sedentary behavior guideline (Meeting ST) were 53.07 and 52.72, respectively. The scores further decreased for groups meeting both the PA and sedentary behavior guidelines (Meeting PA+ST), the sleep guideline (Meeting Sleep), and both the sleep and sedentary behavior guidelines (Meeting Sleep+ST), with average scores of 49.68, 48.44, and 44.75, respectively. The group meeting all 3 guidelines—PA, sleep, and sedentary behavior (Meeting PA+Sleep+ST)—had the lowest score.This effect size indicates that approximately 6% of the variance in SNA scores can be attributed to differences in health behavior patterns (η²=0.06), which is a medium effect according to Cohen's guidelines and may be considered meaningful in behavioral science and mental health research.

### Mental Health Status of University Students With Different 24-HMG Meeting Patterns

**Figure 2.**
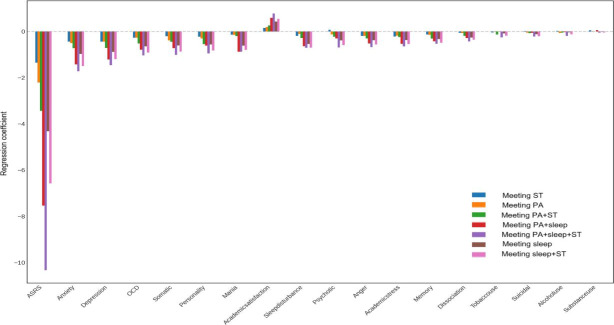
Relationship between different 24-HMG meeting patterns and university students’ mental health. PT: physical activity; ST: sedentary time.

The study results (Figure 2) revealed a significant association between 24-HMG healthy behaviors and mental health status. Among these, simultaneous adherence to the PA, sleep, and sedentary behavior guidelines (Meeting PA+Sleep+ST) demonstrated the strongest and most consistent protective effects, particularly in improving attention deficit (ASRS), depression, and anxiety symptoms. Adherence to both the sleep and sedentary behavior guidelines (Meeting Sleep+ST) and the PA and sleep guidelines (Meeting PA+Sleep) also showed strong protective effects. Except for academic satisfaction, which was positively correlated, all 24-HMG healthy behaviors were negatively correlated with scores for mental health issues among college students. Moreover, composite behaviors (meeting multiple guidelines simultaneously) exhibited stronger protective effects than single behaviors.

### Prediction of the Optimal 24-HMG Behavioral Intervention Strategies for Different Mental Health Issues in University Students With High-Risk of SNA Using Random Forest

This study used a random forest regression model to systematically analyze the relationships between seven 24-HMG healthy lifestyle combinations and 18 mental health variables. Through feature importance ranking, the optimal intervention strategies for each mental health issue were identified. For each mental health variable, cross-validated *R*² scores were calculated, and the feature importance of the seven lifestyle variables was extracted and ranked, generating a detailed report (Multimedia Appendix 3).

**Figure 3.**
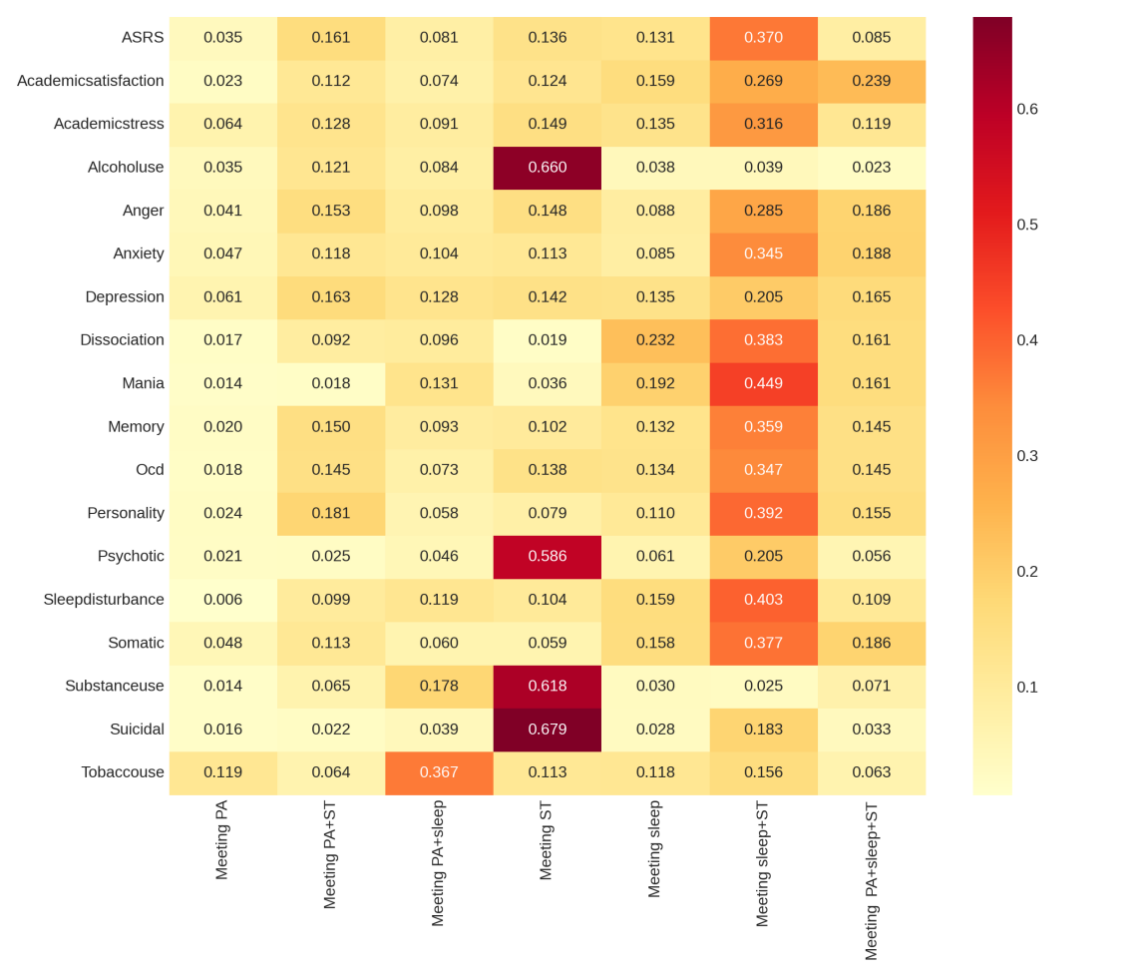
Prediction of optimal 24-hmg guideline health behavior interventions for different mental health issues (This heatmap shows the standard Random Forest feature importance scores; darker colors indicate higher feature importance.)

The analysis results (Figure 3) revealed that Meeting Sleep+ST had high feature importance across most mental health variables, particularly for mania (0.4491), sleep disturbance (0.4032), personality (0.3924), and dissociation (0.3832). The response of different mental health issues to lifestyle interventions varied significantly: Meeting ST had the most significant impact on being suicidal (0.6791) and alcohol use (0.6597), while depressive symptoms were influenced by multiple lifestyle factors, including meeting sleep+ST (0.2053), meeting PA+sleep+ST (0.1650), and meeting PA+ST (0.1634).

Notably, single lifestyle interventions (eg, meeting PA alone or meeting sleep alone) had relatively low importance for most mental health variables, suggesting that combined intervention strategies (especially those involving sleep and screen time) may be more effective than single interventions. The cross-validated *R*² values for all models ranged from 0.0022 to 0.0299, indicating that 24-HMG healthy lifestyle variables had limited explanatory power for mental health variables but still provided some reference value.

By analyzing 4 key metrics—accuracy, precision, recall, and *F*_1_-score (Multimedia Appendix 4)—the results revealed significant differences in the predictive performance across various mental health variables. The ASRS stood out with an accuracy of 0.912, precision of 0.943, recall of 0.911, and an *F*_1_-score of 0.927, indicating that the model performed exceptionally well in identifying and predicting ASRS-related issues. Substance use followed closely, with an *F*_1_-score of 0.873 and balanced metrics (accuracy: 0.856, precision: 0.889, recall: 0.858). Mania ranked third, with an *F*_1_-score of 0.836 and other metrics at accuracy: 0.823, precision: 0.845, and recall: 0.827, demonstrating strong predictive capabilities.

Notably, the precision metric was slightly higher than other metrics for most variables, indicating that the model was cautious in making positive predictions and had a low false positive rate. For example, depression achieved a precision of 0.812, while its recall was 0.789, suggesting that the model prioritized reliability in identifying depressive symptoms. In contrast, some variables, such as memory and somatic, had relatively lower metrics (*F*_1_-scores of 0.756 and 0.742, respectively), which may imply weaker associations between these mental health issues and lifestyle factors or the need to incorporate additional features to improve predictive performance.

Overall, the model demonstrated high reliability in predicting major mental health variables (eg, ASRS, substance use, and mania), with all metrics consistently above 0.8, providing robust support for lifestyle-based mental health risk prediction.

## Discussion

### Principal Findings

This study systematically explored the relationship between social network addiction and mental health among college students, revealing the significant role of healthy behaviors meeting to the 24-HMG in reducing social network addiction risk and improving mental health.

### SNA Risk and Mental Health Issues in College Students

The results indicate that college students at high risk of SNA exhibit significant negative impacts on their mental health. This finding aligns with existing literature, emphasizing that SNA not only adversely affects academic performance and social interactions but also poses a serious threat to mental health. Specifically, the high-risk group scored significantly higher on multiple mental health indicators, such as anxiety, depression, and personality disorders, compared to the low-risk group, providing strong evidence for the causal relationship between SNA and mental health issues.

Recent studies have further validated this connection. For example, Wang et al [50] found, in their cross-sectional study, that students with higher SNA scores had significantly increased mental health risks, particularly in terms of anxiety and depressive symptoms. In addition, Smith and Johnson [51] noted that excessive social network use during the COVID-19 pandemic was positively correlated with the worsening of mental health issues. These results not only highlight the potential harms of SNA, especially during special periods like pandemics, where social networks may amplify psychological stress, but also suggest that environmental and contextual factors of social network use should be considered when designing intervention strategies.

### Relationship Between Meeting 24-HMG Healthy Behaviors and SNA Risk

According to the study data, the low SNA risk group performed better in adhering to the 24-HMG than the high-risk group, particularly in meeting the PA and sleep guidelines. This finding aligns with existing theories and empirical studies, indicating that a healthy lifestyle helps reduce the risk of SNA. Conversely, the high-risk group had lower adherence to the 24-HMG, showing that unhealthy behaviors such as lack of PA, insufficient sleep, and prolonged ST are closely linked to SNA.

These findings suggest that promoting healthy behaviors among college students, such as increasing PA, improving sleep duration, and reducing screen time, may be effective in reducing SNA. A study by Li et al [52] further supports this view, finding that adherence to the 24-HMG guidelines was significantly associated with reduced depression risk, particularly in terms of PA and sleep. In addition, Brown et al [53] noted that comprehensive interventions targeting ST, sleep, and PA within the 24-HMG framework had significant effects on improving mental health. These results indicate that a healthy lifestyle not only helps reduce SNA risk but also significantly enhances mental health.

Notably, simultaneous adherence to the PA, sleep, and sedentary behavior guidelines (Meeting PA+sleep+ST) demonstrated the strongest and most consistent protective effects, particularly in improving attention deficit (ASRS), depression, and anxiety symptoms. This finding underscores the importance of comprehensive intervention strategies, suggesting that multidimensional healthy behaviors should be prioritized in health promotion plans.

### Impact of Meeting 24-HMG Guidelines on SNA

The study results further indicate that as the number of healthy behaviors adhering to the 24-HMG increases, college students’ SNA scores show a gradual decline. The “Meeting None” group, which did not meet any health guidelines, had the highest SNA score, while the “Meeting PA+sleep+ST” group, which simultaneously met the PA, sleep, and sedentary behavior guidelines, had the lowest score. This finding supports the view that increasing participation in healthy behaviors, especially comprehensive health behaviors (eg, combined interventions targeting PA, sleep, and ST), can effectively reduce social network addiction risk.

However, this result also raises deeper considerations regarding the effectiveness of intervention strategies. Although participation in comprehensive health behaviors significantly reduces SNA risk, how to effectively promote these behavioral changes in practical interventions remains a significant challenge. Taylor et al [54] found in their study on children and adolescents with ADHD that adherence to the 24-HMG guidelines was significantly associated with reduced cognitive and social difficulties, further supporting the effectiveness of comprehensive health behaviors in improving mental health and reducing addiction risk. These findings suggest that future intervention strategies should focus on promoting the integration and implementation of healthy behaviors across multiple dimensions.

### Protective Effects of Meeting 24-HMG Guidelines on Mental Health Issues

When exploring the relationship between 24-HMG healthy behaviors and mental health issues, the study found that comprehensive health behaviors (eg, simultaneously meeting the PA, sleep, and sedentary behavior guidelines) had significant protective effects on mental health, particularly in alleviating anxiety and depressive symptoms. In contrast, single health behaviors (eg, increasing PA alone or improving sleep alone) had relatively weaker effects on mitigating mental health issues. This result suggests that in practical interventions, single-behavior interventions may not be sufficient to produce significant mental health improvements.

Therefore, future intervention strategies should focus on composite behavior interventions, encouraging college students to improve multiple lifestyle habits simultaneously to achieve better mental health protection. A study by Anderson et al [55] demonstrated that comprehensive health behavior interventions were highly effective in alleviating anxiety and depressive symptoms, especially during the pandemic. In addition, Miller et al [56] noted that comprehensive lifestyle adjustments have long-term protective effects on mental health. These findings further emphasize the importance of composite behavior interventions, suggesting that multidimensional lifestyle adjustments should be considered when designing interventions to enhance their effectiveness.

### Machine Learning Analysis: Prediction of Optimal Intervention Strategies

This study used a random forest model to analyze the relationship between 24-HMG health behavior combinations and various mental health issues in groups with high-risk SNA, identifying the optimal intervention strategies for different mental health problems. Through feature importance analysis, it was found that meeting the sleep and ST guidelines (Meeting sleep+ST) had a significant protective effect on anxiety, personality disorders, and dissociative symptoms, while the combination of PA and ST (Meeting PA+ST) was most effective in improving depressive symptoms. Although the explanatory power of these lifestyle combinations for mental health in the group with high-risk of SNA has certain limitations, they provide important evidence for developing more personalized and targeted intervention strategies.

In terms of translating these research findings into specific campus interventions, policymakers and university management should enhance cooperation to create practical and feasible campus health policies. For example, implementing mandatory screen time limits, promoting campus-wide digital detox programs, and designing gamified exercise incentive programs could improve students’ engagement in and adherence to healthy behaviors. Harris et al [57] also support the notion that comprehensive lifestyle adjustments significantly improve mental health during the COVID-19 period, indicating that data-driven personalized intervention strategies have strong practical application prospects.

However, while digital intervention tools such as health apps and fitness trackers aid in monitoring and encouraging health behaviors, they are often accompanied by increased screen time, which may lead to negative consequences such as SNA. Future research and practice must balance the benefits of these digital tools with the risks of excessive screen use. Optimizing the design of intervention tools, reducing passive screen time for users, and integrating offline activities are expected to alleviate this paradox and promote positive shifts toward a healthier lifestyle.

### Strengths and Limitations of the Study

The strength of this study lies in its comprehensive and multidimensional analytical approach. First, the large and representative sample (n=12,541) significantly enhances the external validity and generalizability of the study’s findings. Second, an interdisciplinary approach combining psychology, public health, and machine learning techniques facilitated a systematic exploration of the complex relationships between health behaviors and mental health issues. Specifically, the use of a random forest model effectively addressed nonlinear relationships and innovatively enabled personalized intervention predictions based on machine learning, providing precise intervention strategies for different mental health issues. Furthermore, by integrating the 24-HMG health behavior standards, this study constructed a scientifically systematic research framework, deepening the understanding of the relationship between health behaviors and SNA, and laying a solid empirical foundation for future customized interventions.

Although this study holds significant theoretical and practical implications in revealing the relationship between SNA, mental health, and relevant intervention strategies, several limitations must be considered when interpreting the results. First, the cross-sectional design limits the ability to make clear causal inferences. Future research should adopt longitudinal or experimental designs to explore the dynamic causal mechanisms between SNA and mental health. Second, while this study controlled for variables such as age, gender, and academic year, it did not fully account for potential influencing factors such as personality traits, family background, and internet usage patterns. Future studies incorporating multivariate regression analyses with additional covariates would enhance the robustness of causal inferences. Third, all measurements were based on self-reported data, which may be influenced by social desirability bias and recall bias, potentially affecting measurement accuracy and the reliability of the conclusions. Future studies should consider combining objective behavioral measurement tools (eg, PA accelerometers, screen usage logs) to improve data objectivity and research validity, or use actual intervention testing, such as randomized controlled trials, to verify the effectiveness and feasibility of intervention strategies.

Moreover, the sample in this study was primarily drawn from universities in Guizhou Province, China. Although participants were from 12 different provinces, the cultural background, educational policies, and school management practices in the region may influence students’ lifestyle and behavior patterns, which may limit the generalizability of the study’s conclusions. While this study explored the combined effects of various health behaviors such as PA, sedentary behavior, and sleep on SNA, it did not sufficiently analyze the moderating effects of social environmental factors such as cultural background and socioeconomic status, nor did it adequately consider comorbid factors (eg, substance abuse and other psychological disorders) that may influence the results. Future research should integrate these factors’ interactions to gain a more comprehensive understanding of the causes of SNA and its intervention mechanisms.

Finally, since this study focused on Chinese university students, the applicability of the findings across different cultural contexts should be interpreted with caution, given differences in digital usage habits, sleep patterns, and PA levels. Future research targeting Western university student populations will help validate the generalizability of this study and further refine intervention program designs tailored to different cultural backgrounds.

### Conclusion

This study found that university students with high-risk of SNA had significantly higher scores on mental health indicators such as anxiety, depression, and personality disorders compared to students with low-risk, highlighting the serious threat that SNA poses to mental health. The study further revealed that university students who met the 24-HMG health behavior guidelines had significantly better mental health compared to those who did not meet the guidelines, with students who simultaneously adhered to the PA, sleep, and ST guidelines showing stronger effects in alleviating anxiety and depression. Random forest analysis identified the optimal behavioral combinations for different mental health issues, emphasizing the importance of comprehensive intervention strategies.

Although this study provides important theoretical and practical insights, there are some limitations. Given the differences in digital usage habits, sleep patterns, and PA levels across various cultural contexts, the cross-cultural applicability of the study’s results should be interpreted with caution.

## Supplementary material

10.2196/72260 Multimedia Appendix 1 Distribution of Health Behaviors in University Students Adhering to the 24-Hour Movement Guidelines.(The numbers next to the bars represent the sample size (frequency percentage).

10.2196/72260 Multimedia Appendix 2 Comparison of SNAS-C Scores Among College Students Meeting Different 24-HMG Guidelines.

10.2196/72260 Multimedia Appendix 3 Feature Importance Ranking for Optimal 24-HMG Behavioral Interventions Predicted by Random Forest for Different Mental Health Issues.

10.2196/72260 Multimedia Appendix 4 Performance Metrics of the Random Forest Prediction Mode

## Abbreviations

24-HMG: 24-hour movement guidelines
ADHD: attention-deficit/hyperactivity disorder
ASRS: attention-deficit/hyperactivity disorder self-report scale
DSM-5: Diagnostic and Statistical Manual of Mental Disorders, Fifth Edition
DSM-5 CCSM: DSM-5 Self-Report Level 1 Cross-Cutting Symptom Measure for Adults Chinese Version
MRI: magnetic resonance imaging
PA: physical activity
SNA: social network addiction
SNAS-C: social network addiction scale for college students-Chinese version
ST: sedentary time
WHO: World Health Organization

## References

[1] Kuss DJ, Griffiths MD, Karila L, Billieux J (2014). Internet addiction: A systematic review of epidemiological research for the last decade. Curr Pharm Des.

[2] Nazari A, Hosseinnia M, Torkian S, Garmaroudi G (2023). Social media and mental health in students: a cross-sectional study during the Covid-19 pandemic. BMC Psychiatry.

[3] Salari N, Zarei H, Hosseinian-Far A, Rasoulpoor S, Shohaimi S, Mohammadi M (2025). The global prevalence of social media addiction among university students: a systematic review and meta-analysis. J Public Health (Berl).

[4] Andreassen CS, Billieux J, Griffiths MD (2016). The relationship between addictive use of social media and video games and symptoms of psychiatric disorders: A large-scale cross-sectional study. Psychol Addict Behav.

[5] Zhao J, Ye B, Yu L, Xia F (2022). Effects of stressors of COVID-19 on Chinese college students’ problematic social media use: a mediated moderation model. Front Psychiatry.

[6] Wang JL, Wang HZ, Gaskin J, Hawk S (2017). The mediating roles of upward social comparison and self-esteem and the moderating role of social comparison orientation in the association between social networking site usage and subjective well-being. Front Psychol.

[7] Landa-Blanco M, García YR, Landa-Blanco AL, Cortés-Ramos A, Paz-Maldonado E (2024). Social media addiction relationship with academic engagement in university students: The mediator role of self-esteem, depression, and anxiety. Heliyon.

[8] Lin LY, Sidani JE, Shensa A (2016). Association between social media use and depression among US young adults. Depress Anxiety.

[9] Twenge JM, Campbell WK (2018). Associations between screen time and lower psychological well-being among children and adolescents: Evidence from a population-based study. Prev Med Rep.

[10] Sadagheyani HE, Tatari F (2021). Investigating the role of social media on mental health. MHSI.

[11] Andreassen CS, Torsheim T, Brunborg GS, Pallesen S (2012). Development of a Facebook addiction scale. Psychol Rep.

[12] Shahnawaz MG, Rehman U (2020). Social networking addiction scale. Cogent Psychol.

[13] Bi S, Yuan J, Luo L (2024). The social networking addiction scale: translation and validation study among Chinese college students. IJMHP.

[14] Leménager T, Dieter J, Hill H (2016). Exploring the neural basis of avatar identification in pathological internet gamers and of self-reflection in pathological social network users. J Behav Addict.

[15] Kim J ho, Jung S hye, Ahn J chang, Kim B seong, Choi H ju (2020). Social networking sites self-image antecedents of social networking site addiction. J Psychol Afr.

[16] Shin M, Lee J, Chyung YJ, Kim PW, Jung SY (2016). Integrating psychosocial and cognitive predictors of social networking service addiction tendency using structural equation modeling. An International Journal of Psychological Sciences.

[17] He Q, Turel O, Bechara A (2017). Brain anatomy alterations associated with social networking site (SNS) addiction. Sci Rep.

[18] Wadsley M, Ihssen N (2023). A systematic review of structural and functional MRI studies investigating social networking site use. Brain Sci.

[19] Lee D, Lee J, Namkoong K, Jung YC (2021). Altered functional connectivity of the dorsal attention network among problematic social network users. Addict Behav.

[20] Sovani A (2020). Nonsubstance or behavioral addictions: Neuropsychological underpinnings and psychosocial interventions. Ann Indian Psychiatry.

[21] Kwok C, Leung PY, Poon KY, Fung XCC (2021). The effects of internet gaming and social media use on physical activity, sleep, quality of life, and academic performance among university students in Hong Kong: a preliminary study. Asian Journal of Social Health and Behavior.

[22] Alimoradi Z, Lin CY, Broström A (2019). Internet addiction and sleep problems: a systematic review and meta-analysis. Sleep Med Rev.

[23] Guo N, Luk TT, Wang MP (2020). Self-reported screen time on social networking sites associated with problematic smartphone use in Chinese adults: a population-based study. Front Psychiatry.

[24] Brailovskaia J, Margraf J (2020). Relationship between depression symptoms, physical activity, and addictive social media use. Cyberpsychol Behav Soc Netw.

[25] Tremblay MS, Carson V, Chaput JP (2016). Canadian 24-hour movement guidelines for children and youth: an integration of physical activity, sedentary behaviour, and sleep. Appl Physiol Nutr Metab.

[26] Carson V, Hunter S, Kuzik N (2016). Systematic review of sedentary behaviour and health indicators in school-aged children and youth: an update. Appl Physiol Nutr Metab.

[27] Rollo S, Antsygina O, Tremblay MS (2020). The whole day matters: Understanding 24-hour movement guideline adherence and relationships with health indicators across the lifespan. J Sport Health Sci.

[28] Porter CD, McPhee PG, Kwan MY, Timmons BW, Brown DMY (2023). 24-hour movement guideline adherence and mental health: a cross-sectional study of emerging adults with chronic health conditions and disabilities. Disabil Health J.

[29] Ma C, Yan J, Hu H, Shi C, Li F, Zeng X (2022). Associations between 24-h movement behavior and internet addiction in adolescents: a cross-sectional study. Int J Environ Res Public Health.

[30] Ho PTN, Hoepel SJ, Rodriguez-Ayllon M, Luik AI, Vernooij MW, Neitzel J (2024). Sleep, 24-hour activity rhythms, and subsequent amyloid-β pathology. JAMA Neurol.

[31] Heiland EG, Tarassova O, Fernström M, English C, Ekblom Ö, Ekblom MM (2021). Frequent, short physical activity breaks reduce prefrontal cortex activation but preserve working memory in middle-aged adults: ABBaH study. Front Hum Neurosci.

[32] Feter N, Ligeza TS, Bashir N (2024). Effects of reducing sedentary behaviour by increasing physical activity, on cognitive function, brain function and structure across the lifespan: A systematic review and meta-analysis. Br J Sports Med.

[33] Li S, Wu Q, Tang C, Chen Z, Liu L (2020). Exercise-based interventions for internet addiction: neurobiological and neuropsychological evidence. Front Psychol.

[34] Lin LY, Sidani JE, Shensa A (2016). Association between social media use and depression among U.S. young adults. Depress Anxiety.

[35] Wang W, Xie X, Wang X, Lei L, Hu Q, Jiang S (2019). Cyberbullying and depression among Chinese college students: a moderated mediation model of social anxiety and neuroticism. J Affect Disord.

[36] Kessler RC, Adler L, Ames M (2005). The World Health Organization adult ADHD self-report scale (ASRS): a short screening scale for use in the general population. Psychol Med.

[37] Yeh CB, Gau SSF, Kessler RC, Wu YY (2008). Psychometric properties of the Chinese version of the adult ADHD self‐report scale. Int J Methods Psychiatr Res.

[38] Ma SJ, Wang WJ, Tang M, Chen H, Ding F (2021). Evaluation of the construct reliability and validity of the DSM-5 self-rated level 1 cross-cutting symptom measure-Chinese version in maintenance hemodialysis patients. J Int Med Res.

[39] Luo L, Yuan J, Wu C (2025). Predictors of depression among Chinese college students: a machine learning approach. BMC Public Health.

[40] Cohen S, Kamarck T, Mermelstein R (1983). A global measure of perceived stress. J Health Soc Behav.

[41] Diener E, Emmons RA, Larsen RJ, Griffin S (1985). The satisfaction with life scale. J Pers Assess.

[42] Luo L (2024). 24-H movement behaviors and visual impairment among Chinese adolescents with and without obesity. Complement Ther Clin Pract.

[43] Luo L, Cao Y, Hu Y (2022). The associations between meeting 24-hour movement guidelines (24-HMG) and self-rated physical and mental health in older adults-cross sectional evidence from China. Int J Environ Res Public Health.

[44] Luo L, Zeng X, Cao Y (2023). The associations between meeting 24-hour movement guidelines (24-HMG) and mental health in adolescents—cross sectional evidence from China. Int J Environ Res Public Health.

[45] Cohen J (1988). Statistical Power Analysis for the Behavioral Sciences.

[46] Cramer H (1946). Mathematical Methods of Statistics.

[47] Cohen J (1973). Eta-Squared and Partial Eta-Squared in Fixed Factor ANOVA Designs. Educ Psychol Meas.

[48] Tabachnick BG, Fidell LS (2019). Using Multivariate Statistics.

[49] Breiman L (2001). Random forests. Mach Learn.

[50] Wang T, Wong JYH, Wang MP, Li ACY, Kim SS, Lee JJ (2021). Effects of social networking service (SNS) addiction on mental health status in Chinese university students: structural equation modeling approach using a cross-sectional online survey. J Med Internet Res.

[51] Zhao N, Zhou G (2020). Social media use and mental health during the COVID‐19 pandemic: moderator role of disaster stressor and mediator role of negative affect. Appl Psychol Health Well Being.

[52] Zhang Y, Pan Y, Ma Z, Wang D, Zou R, Fan F (2023). Cross-sectional and longitudinal associations of adherence to the 24-hour movement guidelines with mental health problems among Chinese adolescents. J Psychosom Res.

[53] Zhang X, Gu X (2022). Adherence to the 24-hour movement behavior guidelines and associations with depressive symptoms among college students. International Journal of Kinesiology in Higher Education.

[54] Zhao M, Hou M, Herold F (2024). Associations of meeting 24-hour movement behavior guidelines with social and emotional function in youth with ASD/ADHD. J Affect Disord.

[55] Mata J, Wenz A, Rettig T (2021). Health behaviors and mental health during the COVID-19 pandemic: a longitudinal population-based survey in Germany. Soc Sci Med.

[56] Dale H, Brassington L, King K (2014). The impact of healthy lifestyle interventions on mental health and wellbeing: a systematic review. Mental Health Review Journal.

[57] Simjanoski M, Ballester PL, da Mota JC (2022). Lifestyle predictors of depression and anxiety during COVID-19: a machine learning approach. Trends Psychiatry Psychother.

