# Supplementary figures and images for: Mental Health Issues and 24-Hour Movement Guidelines–Based Intervention Strategies for University Students With High-Risk Social Network Addiction: Cross-Sectional Study Using a Machine Learning Approach
Source: J Med Internet Res. 2025 Jun 13;27:e72260. doi: 10.2196/72260 (PMC12180683; doi:10.2196/72260)

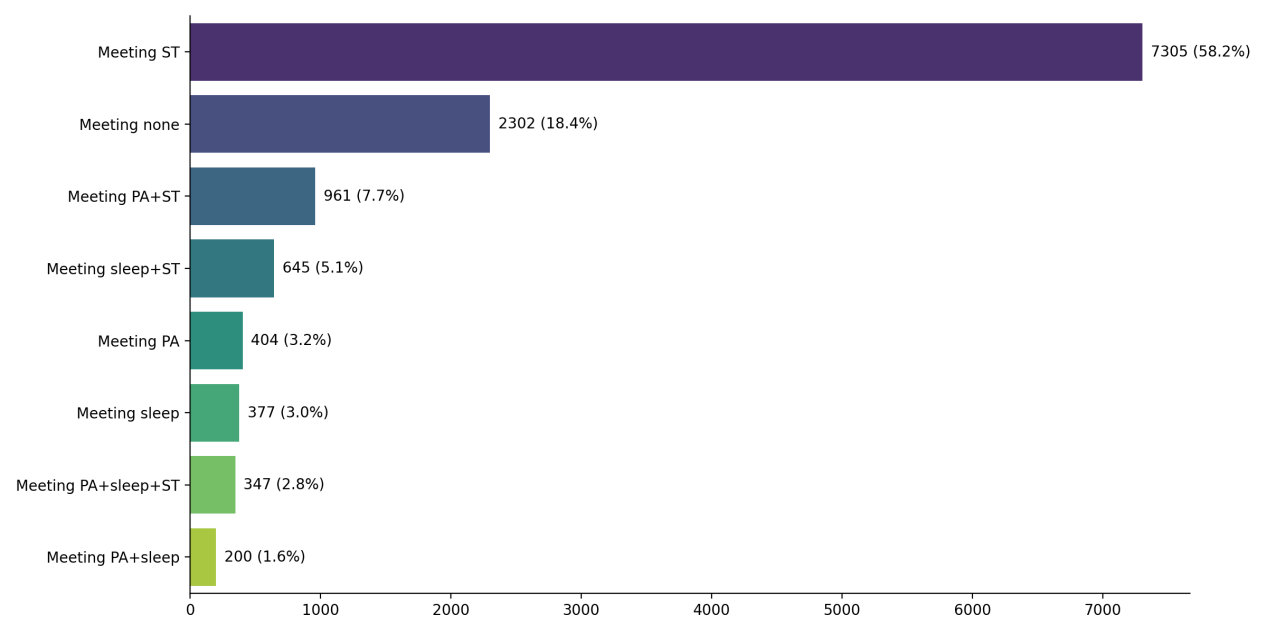

Supplement: Multimedia Appendix 1 [file jmir-v27-e72260-s001.png]

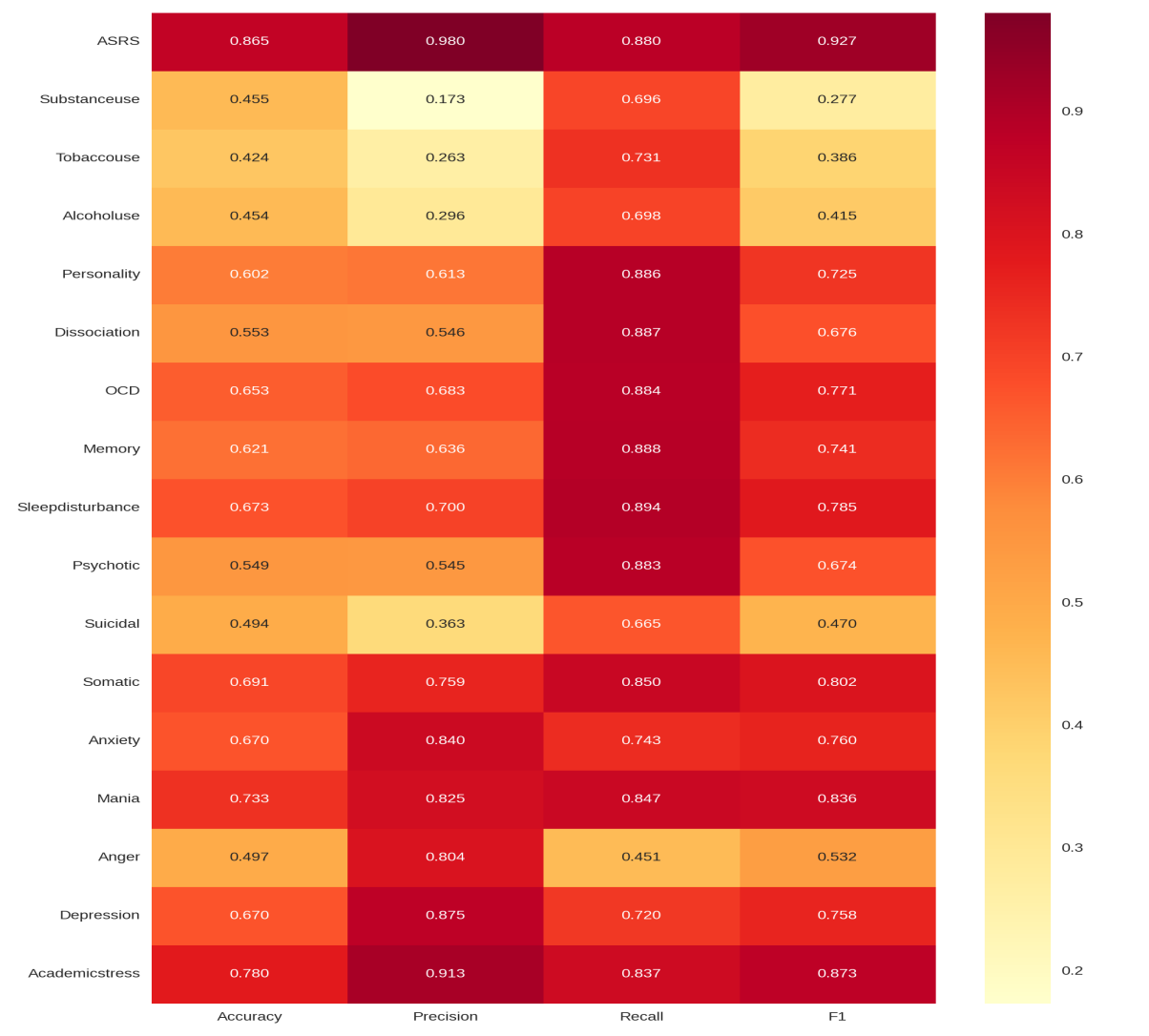

Supplement: Multimedia Appendix 4 [file jmir-v27-e72260-s004.png]
