# Supplementary material for: Mental Health Issues and 24-Hour Movement Guidelines–Based Intervention Strategies for University Students With High-Risk Social Network Addiction: Cross-Sectional Study Using a Machine Learning Approach
Source: J Med Internet Res. 2025 Jun 13;27:e72260. doi: 10.2196/72260 (PMC12180683; doi:10.2196/72260)
Supplement: Multimedia Appendix 2 [file jmir-v27-e72260-s002.docx]

**Appendix 2 .**Comparison of SNAS-C Scores Among College Students Meeting Different 24-HMG Guidelines

| Group | Mean (Standard Deviation) | F-value | *P* value | Effect Size  (*η²*) |
| --- | --- | --- | --- | --- |
| Meeting PA | 53.07（18.47） | 109.80 | 0.00 | 0.06 |
| Meeting PA+ST | 49.68（16.95） | 109.80 | 0.00 | 0.06 |
| Meeting PA+sleep | 43.26（18.84） | 109.80 | 0.00 | 0.06 |
| Meeting PA+sleep+ST | 38.18（16.87） | 109.80 | 0.00 | 0.06 |
| Meeting ST | 52.72（16.19） | 109.80 | 0.00 | 0.06 |
| Meeting none | 57.98（16.35） | 109.80 | 0.00 | 0.06 |
| Meeting sleep | 48.44（18.01） | 109.80 | 0.00 | 0.06 |
| Meeting sleep+ST | 44.75（17.32） | 109.80 | 0.00 | 0.06 |
