# Supplementary material for: Mental Health Issues and 24-Hour Movement Guidelines–Based Intervention Strategies for University Students With High-Risk Social Network Addiction: Cross-Sectional Study Using a Machine Learning Approach
Source: J Med Internet Res. 2025 Jun 13;27:e72260. doi: 10.2196/72260 (PMC12180683; doi:10.2196/72260)
Supplement: Multimedia Appendix 3 [file jmir-v27-e72260-s003.docx]

**Appendix 3.** Feature Importance Ranking for Optimal 24-HMG Behavioral Interventions Predicted by Random Forest for Different Mental Health Issues

| Variable | Meeting ST | Meeting PA | Meeting PA+ST | Meeting PA+sleep | Meeting PA+sleep+ST | Meeting sleep | Meeting sleep+ST | *R^2^* |
| --- | --- | --- | --- | --- | --- | --- | --- | --- |
| Academic satisfaction | 0.124 | 0.023 | 0.112 | 0.074 | 0.239 | 0.159 | 0.269 | 0.017 |
| Academicstress | 0.149 | 0.064 | 0.128 | 0.091 | 0.119 | 0.135 | 0.316 | 0.014 |
| ASRS | 0.136 | 0.035 | 0.161 | 0.081 | 0.085 | 0.131 | 0.370 | 0.003 |
| Alcoholuse | 0.660 | 0.035 | 0.121 | 0.084 | 0.023 | 0.038 | 0.039 | 0.001 |
| Anger | 0.148 | 0.041 | 0.153 | 0.098 | 0.186 | 0.088 | 0.285 | 0.020 |
| Anxiety | 0.113 | 0.047 | 0.118 | 0.104 | 0.188 | 0.085 | 0.345 | 0.021 |
| Depression | 0.142 | 0.061 | 0.163 | 0.128 | 0.165 | 0.135 | 0.205 | 0.030 |
| Dissociation | 0.019 | 0.017 | 0.092 | 0.096 | 0.161 | 0.232 | 0.383 | 0.008 |
| Mania | 0.036 | 0.014 | 0.018 | 0.131 | 0.161 | 0.192 | 0.449 | 0.008 |
| Memory | 0.102 | 0.020 | 0.150 | 0.093 | 0.145 | 0.132 | 0.359 | 0.015 |
| OCD | 0.138 | 0.018 | 0.145 | 0.073 | 0.145 | 0.134 | 0.347 | 0.014 |
| Personality | 0.079 | 0.024 | 0.181 | 0.058 | 0.155 | 0.110 | 0.392 | 0.010 |
| Psychotic | 0.586 | 0.021 | 0.025 | 0.046 | 0.056 | 0.061 | 0.205 | 0.004 |
| Sleep disturbance | 0.104 | 0.006 | 0.099 | 0.119 | 0.109 | 0.159 | 0.403 | 0.030 |
| Somatic | 0.059 | 0.048 | 0.113 | 0.060 | 0.186 | 0.158 | 0.377 | 0.012 |
| Substanceuse | 0.618 | 0.014 | 0.065 | 0.178 | 0.071 | 0.030 | 0.025 | 0.004 |
| Suicidal | 0.679 | 0.016 | 0.022 | 0.039 | 0.033 | 0.028 | 0.183 | 0.004 |
| Tobaccouse | 0.113 | 0.119 | 0.064 | 0.367 | 0.063 | 0.118 | 0.156 | 0.002 |
